# Human regulatory memory B cells defined by expression of TIM-1 and TIGIT are dysfunctional in multiple sclerosis

**DOI:** 10.3389/fimmu.2024.1360219

**Published:** 2024-04-30

**Authors:** Johnna F. Varghese, Belinda J. Kaskow, Felipe von Glehn, Junning Case, Zhenhua Li, Amélie M. Julé, Emma Berdan, Shannan Janelle Ho Sui, Yong Hu, Rajesh Krishnan, Tanuja Chitnis, Vijay K. Kuchroo, Howard L. Weiner, Clare Mary Baecher-Allan

**Affiliations:** ^1^Harvard Medical School, Boston, MA, United States; ^2^Ann Romney Center for Neurologic Diseases, Department of Neurology, Brigham and Women’s Hospital, Boston, MA, United States; ^3^Bioinformatics Core, Department of Biostatistics, Harvard T.H. Chan School of Public Health, Boston, MA, United States; ^4^The Gene Lay Institute of Immunology and Inflammation, Harvard Medical School, Brigham and Women’s Hospital, Boston, MA, United States

**Keywords:** regulation, multiple sclerosis, Breg, TIM-1 B cells, TIGIT

## Abstract

**Background:**

Regulatory B cells (Bregs) play a pivotal role in suppressing immune responses, yet there is still a lack of cell surface markers that can rigorously identify them. In mouse models for multiple sclerosis (MS), TIM-1 or TIGIT expression on B cells is required for maintaining self-tolerance and regulating autoimmunity to the central nervous system. Here we investigated the activities of human memory B cells that differentially express TIM-1 and TIGIT to determine their potential regulatory function in healthy donors and patients with relapsing-remitting (RR) MS.

**Methods:**

FACS-sorted TIM-1+/-TIGIT+/- memory B (memB) cells co-cultured with allogenic CD4+ T cells were analyzed for proliferation and induction of inflammatory markers using flow cytometry and cytokine quantification, to determine Th1/Th17 cell differentiation. Transcriptional differences were assessed by SMARTSeq2 RNA sequencing analysis.

**Results:**

TIM-1-TIGIT- double negative (DN) memB cells strongly induce T cell proliferation and pro-inflammatory cytokine expression. The TIM-1+ memB cells enabled low levels of CD4+ T cell activation and gave rise to T cells that co-express IL-10 with IFNγ and IL-17A or FoxP3. T cells cultured with the TIM-1+TIGIT+ double positive (DP) memB cells exhibited reduced proliferation and IFNγ, IL-17A, TNFα, and GM-CSF expression, and exhibited strong regulation in Breg suppression assays. The functional activity suggests the DP memB cells are a bonafide Breg population. However, MS DP memB cells were less inhibitory than HC DP memB cells. A retrospective longitudinal study of anti-CD20 treated patients found that post-treatment DP memB cell frequency and absolute number were associated with response to therapy. Transcriptomic analyses indicated that the dysfunctional MS-derived DP memB/Breg population exhibited increased expression of genes associated with T cell activation and survival (CD80, ZNF10, PIK3CA), and had distinct gene expression compared to the TIGIT+ or TIM-1+ memB cells.

**Conclusion:**

These findings demonstrate that TIM-1/TIGIT expressing memory B cell subsets have distinct functionalities. Co-expression of TIM-1 and TIGIT defines a regulatory memory B cell subset that is functionally impaired in MS.

## Introduction

Several mechanisms have been identified that contribute to chronic central nervous system (CNS) inflammation in MS (1). It has been well established that CNS autoreactive T helper Th1 and Th17 T cells exhibit pathogenic effector activity in MS (2). The high efficacy of B cell-depleting anti-CD20 monoclonal antibody therapies is associated with the potent antigen presenting (APC) capacity of B cells to activate pathogenic T cells via surface protein interaction and cytokine production (3). B cells from MS patients exhibit higher surface expression of HLA-DR, CD40, and CD80/86 than healthy donor (HC) derived B cells suggesting that B cells may be highly poised towards antigen presentation in the MS patients (4) with CD40, CD80, and CD86, also contributing towards significant genetic risk (5). Additionally, depletion of CD20+ B cells significantly alters the profiles of the circulating T cells with a reduction in IFNγ and IL-17A expression and diminished T cell differentiation (6–8). Furthermore, MS-derived B cells have been shown to directly engage in pathogenic APC activity via their capacity to induce brain-homing, CNS-autoreactive Th1 cells to undergo HLA-Class II-dependent, auto-proliferation in the absence of exogenous antigen (9). Subsets of these T cell activating, pro-inflammatory B cells have been described to express high levels of GM-CSF and/or IL-6 and be increased in frequency in MS versus HC peripheral blood (10, 11).

Regulatory B cells (Bregs) are fundamental in preventing autoimmunity and transplant rejection by suppressing the proliferation and differentiation of effector T cells (1). Historically, IL-10 expression has been used as a hallmark of Breg activity, though Bregs can also regulate immune responses via the expression of inhibitory molecules: PD-L1, FasL, IL-35, or TGFβ (12). In humans, Breg identification is difficult due to a lack of cell surface markers that can faithfully identify them. An exception is transitional B cells that at their point of maturation, can be defined by high expression of CD24 and CD38 (CD19+CD24^hi^CD38^hi^) and exert regulatory function even though these cells ultimately give rise to non-regulatory B cells (13). Other B cell populations that show regulatory capacity are CD19+CD24^hi^CD27+ B cells (B10 Bregs) (14), PD-L1+ B cells (PDL1+Bregs), Granzyme B+ B cells (GzmB+Bregs), and innate-like IL-27-producing i27-Bregs that comprise ~30% of CD20+CD27+CD43+CD11b+ B1 cells (15, 16). The frequency of transitional and IL-10-producing B cells was shown to be reduced in MS compared to HC (17) but were increased following IFN-β therapy (18–20). Therapeutic response to Fingolimod and Siponimod was also associated with an increase in transitional, i27, and TGFβ+ B cells (21, 22). However, since Bregs primarily need an operational definition, it has been challenging to determine the frequency and function of Bregs in patient vs healthy donor studies.

Specific Breg populations are required to maintain CNS homeostasis (23, 24). In the mouse model of MS, experimental autoimmune encephalomyelitis (EAE), the transfer of IL-10+ Bregs can normalize immune cells in the CNS, activate oligodendrocyte precursors, and induce remyelination (25). Mice generated to specifically lack regulatory molecules including TGFβ, IL-35, or Integrin α4 in B cells exhibit severely worse disease upon EAE induction (26–28). T cell Ig and mucin domain protein 1 (TIM-1) was identified as a surface marker for Bregs in mice as it is expressed on all IL-10 producing Bregs. Genetic deletion of TIM-1 in B cells resulted in spontaneous multi-organ tissue inflammation, splenomegaly, lymphadenopathy and spontaneous paralytic disease characterized by CNS inflammation (29, 30). TIM-1+ Bregs also express multiple immune checkpoint molecules, including TIGIT. Mice with conditional deletion of TIGIT in B cells (TIGIT^BKO^) resulted in the development of spontaneous CNS disease but unlike TIM-1^BKO^, the TIGIT^BKO^ mice exhibited less severe tissue inflammation in the peripheral organs (29). Together these data suggest that co-expression of TIM-1 and TIGIT in B cells is required to provide optimal CNS immune regulation.

Here we examined the regulatory capacity of human B cells that express TIM-1 and/or TIGIT and whether these cells are dysfunctional in MS patients. Since it has been proposed that Bregs may arise from the terminal differentiation of memory B (memB) cells (31), we specifically excluded transitional B cells and naïve B cells, which both exhibit regulatory activity. We tested B cell subsets isolated from HCs, RRMS patients before and after anti-CD20 therapy, and RRMS patients that were on natalizumab (anti-VLA4 integrin that binds to fibronectin/VCAM to cross the BBB) therapy. Untreated patients are increasingly rare and natalizumab therapy represents a suitable population to study the immune system in MS as the blocking of leukocytes into the CNS increases the frequency of immune populations in the peripheral blood including the potentially pathogenic and regulatory cells that would otherwise have entered the CNS (32, 33). Surprisingly we found that although the TIM-1+ (TIGIT-) memory B cells do not promote T cell activation unlike the TIM-1-TIGIT- memory B cells, the cells co-expressing TIM-1 and TIGIT represent a potent Breg population that lack both T cell stimulating activity and actively suppress T cell proliferation and Th1/Th17 differentiation. However, when isolated from patients with RRMS (Natalizumab treated), both the TIM-1+ and TIM-1+TIGIT+ B cells exhibit unique functional defects with reduced regulatory activity. Here, we present the functional activities and unique gene expression profiles of memory CD19+ CD20+ CD24^low^ CD38^low^ CD27+ B cells that exhibit distinct *ex vivo* expression of TIM-1 and TIGIT.

## Materials and methods

### Patients

Patient samples were from the outpatient MS clinic at the Brigham and Women’s Hospital (BWH). All the patients belong to the Comprehensive Longitudinal Investigation in MS at BWH Study (The CLIMB Study). Peripheral blood was obtained from RRMS patients and age- and sex-matched healthy volunteers with signed informed consent as approved by the Institutional Review Board of Brigham and Women’s Hospital. All MS patient samples used for these analyses were from patients being treated with Natalizumab except for those described as MS-R (Rituxan), or the pre-/post-anti CD20 treated patients. Pre-anti-CD20 refers to samples taken from patients previously treated with non-anti-CD20 disease-modifying therapies with no drug washout period immediately before their first dose of anti-CD20. Post-anti-CD20 refers to the blood sample taken at their 6-month follow-up appointment before the second dose of anti-CD20. The different therapies were included in the matching of patients with increased vs decreased EDSS cohorts, and are shown in **Supplementary Tables 2**, **3**).

### Cell sorting

Peripheral blood mononuclear cells (PBMCs) were isolated by Ficoll-Hypaque (GE Healthcare Biosciences, UK) gradient centrifugation, cryopreserved in fetal bovine serum (FBS) containing 10% dimethyl sulfoxide and stored in liquid nitrogen until use. Total CD19+ B cells were isolated from cryopreserved PBMCs using CD19+ positive magnetic bead isolation (Miltenyi Biotec, Auburn, CA). Purified B cells were stained with viability dye eFluor™ 506 (Invitrogen, Waltham, MA) in phosphate buffer saline (PBS) for 10 mins at room temperature (RT), washed, followed by incubation with FcR Block (BioLegend, San Diego, CA) for 15 mins at 4°C. The cells were then incubated with fluorochrome-labelled monoclonal antibodies against CD19 PE (clone HIB19), CD20 Pacific Blue (clone 2H7), CD24 PerCP-Cy5.5 (clone ML5), CD38 AF700 (clone HB-7), CD27 FITC (clone H-T27I), TIM-1 APC (clone ID12), and TIGIT PeCy7 (Clone A15153G) all obtained from BioLegend (San Diego, CA) for 30 mins at 4°C. Cells were washed and sorted on FACS Aria III (Becton Dickenson Biosciences, San Diego, CA) 70 uM filter, 50 psi. Population purity was >95%. The absolute cell count of each B cell subtype was calculated based on the volume of blood obtained from each donor.

### CD4+ T cell isolation and labelling

To obtain a large stock of allogenic CD4+ T cells to use as a standard responding T cell for all assays, total CD4 T cells were isolated from a Leukopak (BWH Clinical Labs) using a CD4+ negative isolation kit (Miltenyi Biotec, Auburn, CA). Purity was checked by analyzing CD4+ expression on flow cytometry (>95%). CD4+ T cells were labelled using 0.25μM cell trace violet (CTV) as described (34) and stock vials were prepared and stored in liquid nitrogen until further use.

### Cell culture reagents and *in vitro* microculture assay (B-T and B-B-T)

B and T cells were cultured in RPMI 1640 media supplemented with 5% heat-inactivated FBS (Gibco Life Technologies, Waltham, MA), 1M HEPES, 1mM sodium pyruvate, 1X non-essential amino acids and 1X penicillin/streptomycin. Cells were cultured with 1μg/ml of soluble anti-CD3 (clone Hit3A) (Fisher Scientific, Waltham, MA). For B-T Assays, each B cell subset was cultured at a 1:2 ratio with CTV-labelled allogenic CD4+ T cells. For B-B-T assays, TIM-1+ memB (TIM-1+ TIGIT-), TIGIT+ memB (TIM-1- TIGIT+) or double positive (DP) memB (TIM-1+TIGIT+) cells were co-cultured with autologous double negative (DN) memB (TIM-1-TIGIT-) cells and allogenic CTV-labelled CD4+ T cells at a ratio of 1:1:2. All cultures were incubated at 37°C with 5% CO_2_ and 95% humidity for 6 days. Patient cohort demographics are provided in **Supplementary Table 1**.

### Flow cytometry analysis

Following 6 days of co-culture 100μL of supernatant was collected and stored at -20°C until analysis. The cells were labelled with fixable viability dye eFluor™ 506 for 10 minutes at RT, washed followed by a 2 hr stimulation with leukocyte activation cocktail containing golgi plug (Fisher Scientific, Waltham, MA) at 37°C with 5% CO_2_ and 95% humidity. The cells were fixed for 1 hr at 4°C with the FoxP3 staining kit (Fisher Scientific, Waltham, MA), permeabilized for 15 min at 4°C with blocking from normal rat serum followed by staining for CD4 FITC (clone RPA-T4), CD19 APC-Cy7 (clone HIB19), IL-17A BV786 (clone BL168), IFNγ AF700 (clone 4S.B3), IL-10 AF647 (clone JES3-9D7), FoxP3 PeDazzle (clone 206D), GM-CSF PE (clone BVD2-21C11), TNFα BUV395 (clone MAb11) all from BioLegend (San Diego, CA). Data were acquired using FACS Symphony or BDFortessa (BD Biosciences) and analyzed using FlowJo10 software (TreeStar Industries, Ashland, OR).

### Supernatant cytokine quantification

The Legendplex™ CD8/NK Panel (Human (BioLegend Cat No. 740267) was used to determine the levels of specific analytes; IL-4, IL-10, IL-6, IL-17A, TNFα and IFNγ. The assay was performed as per the manufacturer’s protocol. The assay was run on a BD LSR II FACS instrument. The assay lower limit detection for IL-10 was 20pg/ml and the upper limit for IFNγ was 4000 pg/ml based on standard curves run for each experiment.

### Smart-Seq2 RNA sequencing, data analysis and qPCR validation

1000 cells of each TIM-1/TIGIT expressing memory B cells were sorted and collected in RNAse/DNAse free eppendorf tubes containing 5μl of TCL buffer (Qiagen, Hilden, Germany) along with 1% 2-mercaptoethanol (BME) (Gibco, Life Technologies, Waltham, MA). The sorted cells were transferred to an Eppendorf twin.tec LoBind skirted 96-well PCR plate (Eppendorf, Hamburg, Germany) and protectively sealed using Microseal ‘F’ Foil (BioRad Laboratories, Hercules, CA). The plates were stored at -80°C and processed by the BROAD Institute Genomics platform for Smart-Seq2 RNA-seq. The Trombetta protocol was used sequentially to clean up the lysate, reverse transcribe the mRNA, amplify the transcriptome, PCR clean up, Nextera XT sequencing-library construction, DNA SPRI bead cleanup, and 2 × 38bp paired sequencing (35). Illumina NextSeq500 was used to run the sequencing at the BROAD Genomics platform, (BROAD Institute of MIT and Harvard). The data was demultiplexed and delivered in FASTQ format. Sequencing reads were quality-controlled, mapped and quantified using the bcbio-nextgen bulk RNA-seq pipeline (version 1.2.8-1c563f1). Specifically, reads were aligned to the NCBI build hg38 of the human genome (Homo sapiens) using STAR (version 2.6.1d) (36). Quality control (QC) metrics from FastQC (version 0.11.8), Qualimap (version 2.2.2d) and Samtools (version 1.9) were summarized with MultiQC (version 1.9) (37). Samples with fewer than 4 million total mapped reads, with a low mapping rate to exonic regions of the genome (<25%), or with high mapping rates to either rRNA or intergenic regions (>40%) were initially excluded from the analysis (4 healthy control samples, 7 MS samples). However, subsequent analysis indicated that we were underpowered. Thus, we revisited and reviewed global patterns in the transcriptomic data using principal component analyses (PCA). If QC outliers were PC outliers, we continued excluding them from the analysis (all 4 healthy control samples and 3 MS samples) and retained the others (4 MS samples). Transcripts were quantified through a quasi-alignment approach using Salmon (version 0.14.2) (38). Transcript-level counts were aggregated at the gene level and imported into R using tximport (39), with transcript-level information as well as gene names and biotypes sourced from Ensembl release 94. The resulting count matrix was filtered so that only protein-coding genes with at least 10 total reads detected across all samples were considered in downstream analyses.

Differential expression analyses (DEA) on the remaining samples were performed in R (version 4.2.1) using DESeq2 (version 1.38.3) (40). Only genes with a count of 10 or higher in at least 20% in one of the two contrast groups (e.g., HC and MS TIM-1+TIGIT+ memB (Breg)) were considered in the analysis. Pairwise comparisons were performed between HC and MS derived TIM-1+TIGIT+ memB (Breg) population, between the HC TIM-1+ or HC TIGIT+ memB and the HC DP (Breg) population, and between the MS TIM-1+ or MS TIGIT+ memB and the MS DP (Breg) population. For comparisons of DP Breg samples vs. TIM1+, HC and MS samples were analyzed separately with models including cell population and individual ID. The MS model additionally included rRNA rate as a covariate as initial analyses indicated that this was a significant covariate. Similarly, for comparison of DP Breg vs. TIGIT+ cells, separate models for HC and MS included cell population, rRNA rate, and individual ID. For comparison of HC vs. MS in the DP Breg population, the model included disease status and exonic mapping rate. Statistical significance for all models was determined using a minimum absolute log_2_ fold change of 0.5 and a Benjamini-Hochberg adjusted p-value < 0.1.

For subsequent functional analyses and data visualization, DESeq2-derived log_2_ fold change (LFC) values were corrected using the apeglm shrinkage estimator (41) and count matrices were normalized using log_2_ transformation. Heatmaps were generated using pheatmap (version 1.0.12) and ComplexHeatmap (version 2.14.0). Volcano plots were created using EnhancedVolcano 1.16. To determine if related genes showed differential expression trends we used Gene Set Enrichment Analysis (GSEA). We ranked our genes by log_2_ fold-change and ran a GSEA enrichment test using the MsigDB hallmark database. Statistical significance was determined using a Benjamini-Hochberg adjusted p-value < 0.05.

For qPCR validation of RNA-Seq DGE, the cells were directly sorted into RLT RNA isolation buffer. RNA was isolated using the Qiagen Plus Micro Kit (Qiagen, Netherlands) and converted to cDNA via reverse transcriptase by random hexamers and *MuLV* transcriptase (Applied Biosystems, Foster City, CA). Samples were subjected to real-time PCR analysis on the PRISM 7000 Sequencer Detection System (Applied Biosystems) under standard conditions. Values are represented as the difference in Ct values normalized to *β2-microglobulin* for each sample as per the following formula: relative RNA expression = (2^−dCt^) × 10^3^.

### Statistical analysis

Cellular Data was analyzed using GraphPad Prism Software (Version 9) and results are represented as (mean ± SEM). Significance was determined by one-way ANOVA with correction for multiple tests (Sidak’s multiple comparisons). A p-value <0.05 was considered statistically significant. Gene expression data were analyzed as discussed above.

## Results

### The frequencies of memory B cells that express TIM-1 and/or TIGIT remain constant with therapy

**Figure 1:**
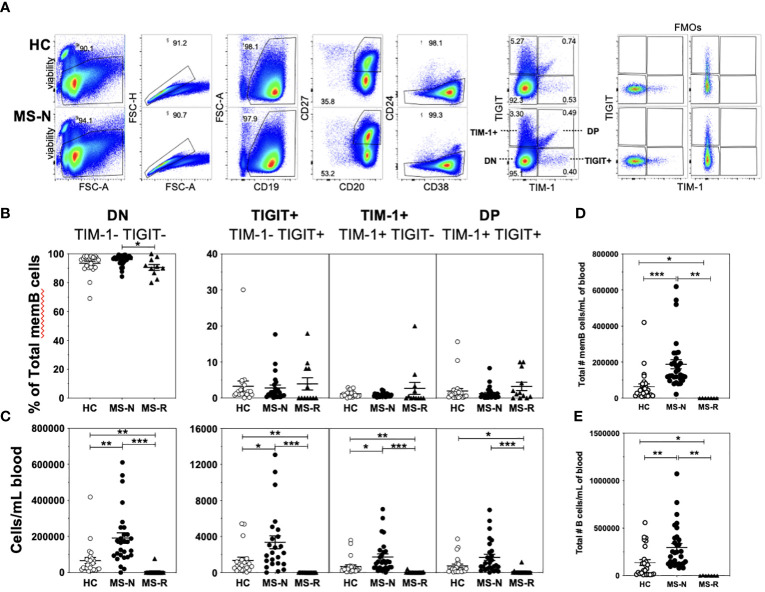
A low frequency of circulating human memB cells express TIM-1 and/or TIGIT. **(A)** Cell sorting and analysis strategy to identify memory B cells from CD19-bead enriched B cells that differentially express TIM-1 and/or TIGIT in HC and MS. **(B)** The frequency of TIM-1+(TIGIT-), TIGIT+ (TIM-1-), TIM-1-TIGIT- (DN), and TIM-1+TIGIT+ (DP) memory B cells was determined as the frequency of the memory B cell population for PBMCs from HCs (open circles- HC), and RRMS patients that were treated with Natalizumab (filled circles, MS-N) or Rituximab (filled triangles, MS-R). The representation of each of these populations per ml of blood was determined. **(C)** The number of cells per each population per mL of blood was determined. The per mL number of **(D)** total memB cells and total number of **(E)** total B cells were also determined. Significance was determined by unpaired Brown-Forsythe and Welch ANOVA tests, with Dunnett’s T3 multiple comparisons test. (*p<0.05, **p<0.005, ***p<0.0005).

Four populations of memory B cells defined by surface expression of TIM-1 and TIGIT were quantified in CD19+CD20+CD27+CD38+/-CD24^med/lo^ B cells after specifically excluding the transitional B cell subset (CD38+CD24^hi^) by flow cytometry of both HC and MS samples (gating shown in **Figure 1A**, showing individual FMOs for TIM-1 and TIGIT). We refer to the four FACS-sorted memory B cell (memB) populations as double negative (DN, TIM-1-TIGIT-), TIM-1+ (TIM-1+TIGIT-), TIGIT+ (TIM-1-TIGIT+) and double positive (DP, TIM-1+TIGIT+) cells. First, we determined whether the frequencies of these B cell subsets within the memB cell pool were altered in MS patients following treatment with anti-CD20 (Rituximab, MS-R) or anti-CD49d (Natalizumab, MS-N) (**Figure 1B**) compared to HCs. Surprisingly, we found that except for the MS-derived DN memB cells whose frequency was lower after anti-CD20 treatment, the relative frequencies of the TIM-1/TIGIT expressing memB cell subsets within the remaining memB cell populations did not differ after the respective treatments. However, as these two treatments greatly affect the level of circulating B cells, we also investigated whether these memB cells exhibited differential representation in the circulation. In examining the absolute number of each B cell subset per ml of blood (**Figures 1C–E**), we found that all B cell subsets were significantly lower in the anti-CD20 treated (MS-R) MS patient samples as expected. In contrast, in analyzing samples from Natalizumab-treated patients, which blocks CD49d-mediated migration of immune cells to the CNS, the MS-N samples were significantly enriched for all memB subsets except for the DP memB cells compared to the HC samples.

### DP and TIM-1+ memB cells are functionally distinct from the pro-inflammatory DN and TIGIT+ memB cell populations

**Figure 2:**
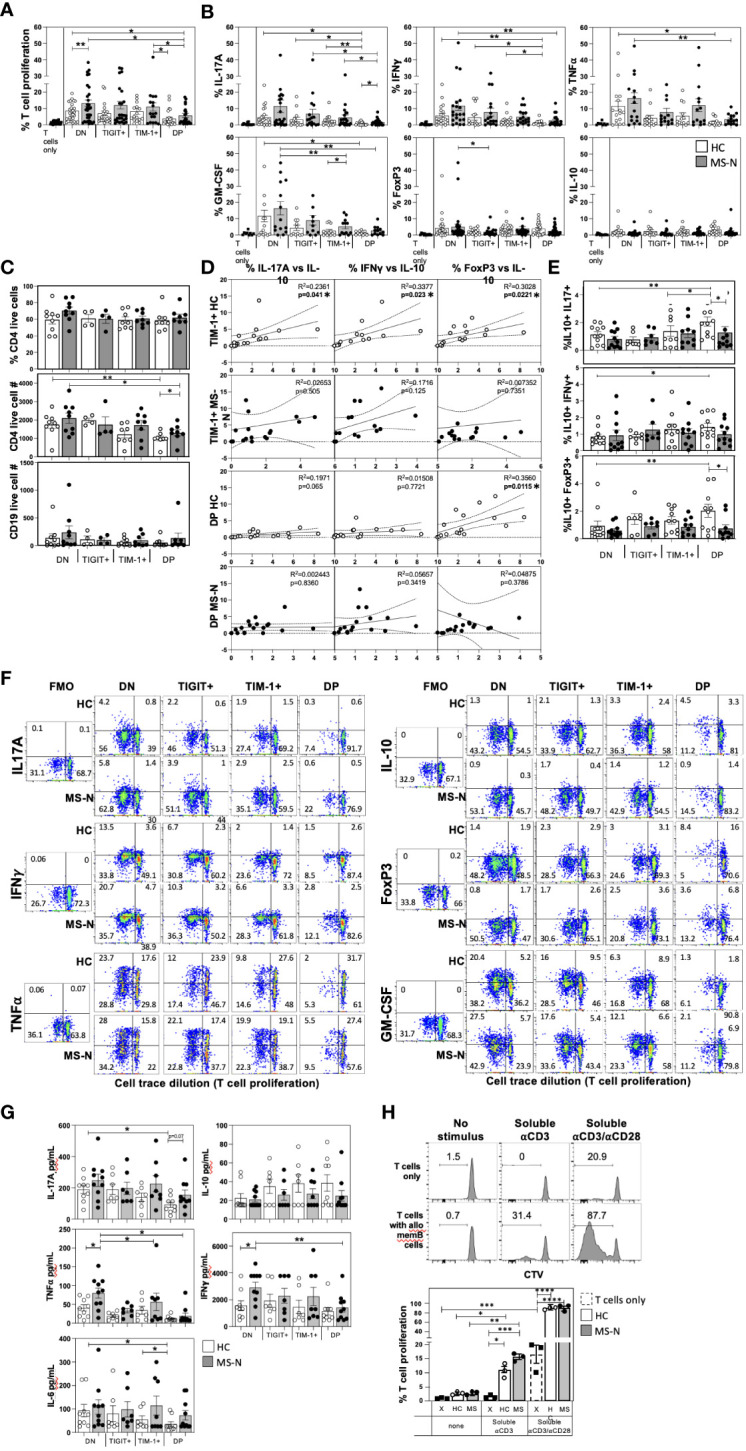
MS and HC TIM-1+/-TIGIT+/- memB cells differentially activate co-cultured T cell proliferation and cytokine expression. The capacity of each DN, TIM-1+, TIGIT+, and DP memB cell population to promote the activation of uniform allogenic CD4+ T cells was determined by co-culture at a 1:2 ratio in the presence of soluble anti-CD3 stimulation. The CD4+ T cells were all from the same HC donor. All MS donors were being treated with Natalizumab. The different HC or MS-derived memB cell subsets exhibited distinct abilities to promote **(A)** T cell proliferation (measure by cell trace violet dilution), and **(B)** Intracellular T cell expression of IL-17A, IFNγ, GM-CSF, TNFα, IL-10 and FoxP3. **(C)** The percent of viable T cells (CD4+e506- out of total CD4+ cells), and the number of viable CD4+ and CD19+ cells in each culture well were determined. **(D)** We correlated the levels of cells that express IL-10 with the percent of T cells expressing IFNγ, IL-17A or FoxP3 in cultures established with the TIM-1+ memB cells or with TIM-1+TIGIT+ DP memB cells. MS are filled circles, HC are open circles. **(E)** The frequency of T cells co-expressing IL-10 and IFNγ, IL-17A or FoxP3 was determined. **(F)** Representative FACS plots for each analyte detected in the different memB subset B-T cultures is shown comparing HC and MS samples. An FMO is shown to determine gating. **(G)** Secreted cytokines were detected by BioLegend Legendplex bead-based ELISA. **(H)** Comparison of allogeneic effects of day 6 co-cultures of the 3^rd^ party HC CD4+ T cells with HC or MS FACS-sorted memB cells. The T cells were cultures with or without DN memB cells with either no additional stimulus, soluble anti-CD3, or soluble anti-CD3/anti-CD28. Within each donor type, significance was determined by paired One-Way Anova and HC vs. MS comparison by unpaired One-way Anova, Sidak’s correction for multiple comparisons was used for all analyses. (*p<0.05, **p<0.005, ***p<0.0005 and ****p<0.0001).

We next examined the capacity of the TIM-1/TIGIT memB cell populations to support T cell activation with expression of Th1 (IFNγ, TNFα) or Th17 (IL-17A) cytokines, or to induce T cells with regulatory potential by inducing expression of IL-10 or FoxP3. The four memB cell populations were FACS-sorted from MS-N and HC PBMCs and co-cultured with third-party HC-derived CD4+ T cells at a 1:2 (B-T cell) ratio to measure their relative ability to promote T cell proliferation and cytokine expression. Although not antigen-specific, the assay was designed to use a weak soluble anti-CD3 stimulus that requires the presence of a cell with antigen-presenting ability and co-stimulatory activity to induce TCR signaling (42). To avoid potential confounding effects of heterogeneous patient-derived T cells, the assay was also established to be allogenic, where all B cell subsets are tested for their ability to activate the identical, HC-derived CD4+ T cells (to negate sample differences in Th1/Th17 precursors). The B-T assay was cultured for 6 days before assaying the viable CD4+ T cells in the different memB co-cultures for proliferation (**Figure 2A**) and intracellular expression of cytokines and FoxP3 (**Figure 2B**).

The different MS or HC-derived TIM-1/TIGIT memB cell populations promoted strikingly different levels of T cell proliferation, Th1/Th17 cytokines, and FoxP3 expression (**Figures 2A, B**). The DN memB cells consistently induced the highest level of T cell proliferation and expression of IFNγ, IL-17A, TNFα and GM-CSF compared to the other memB cell subsets within each donor group (MS or HC). Furthermore, the MS DN memB cells induced greater T cell proliferation than the HC DN memB cells. Surprisingly, the TIGIT+ memB cells also promoted T cell proliferation and cytokine production at levels approaching that induced by the DN memB cells, however, unlike the DN memB cells, the MS-derived TIGIT+ memB cells did not induce T cell FoxP3 expression. The TIM-1+ memB cells from both HC and MS did not differ from each other or from their respective DN memB cells in their ability to induce T cell proliferation or IL-17A and TNFα expression. Yet, the MS TIM-1+ memB cells induced greater T cell expression of GM-CSF and IFNγ than the HC TIM-1+ memB cells. Lastly, the TIM-1+ TIGIT+ DP memB cells showed the lowest cytokine-inducing activity of all subsets. Both HC and MS-N DP memB cell co-cultures gave rise to T cells exhibiting low proliferation and reduced expression of TNFα, IFNγ and GM-CSF, although only the HC DP memB cell co-cultures showed a reduction in expression of IL-17A compared to MS DP memB. Thus, although both the HC and MS DP memB cells poorly supported the activation of T cells, the T cells that did grow with the MS DP memB cells were markedly enriched for IL-17A expression.

Since the different B cells could also potentially induce T cell death, the B-T co-cultures were also examined for viability and cell counts. We found no differences in CD4+ T cell viability or live B cell counts while changes in live CD4+ T cell counts were only detected in the co-cultures that induced disparate levels of T cell proliferation (**Figure 2C**). No memB cell subset increased the frequency of IL-10+ T cells or IL-10 secretion. However, as T cells co-expressing IL-10 with IFNγ or IL-17A are less pathogenic in MS (43, 44), we assessed whether HC and MS-derived TIM-1+ or DP memB cells induced increased expression of IL-10 in direct accordance with the levels of induced pro-inflammatory IFNγ or IL-17 (**Figure 2D**). Only the HC-derived TIM-1+ memB cells produced T cells that expressed higher IL-10 with increases in IFNγ, IL-17A, or FoxP3 expression, and only the HC-derived DP memB cells produced T cells that showed increases in FoxP3 and IL-10 expression (**Figure 2D**). We next directly assessed whether these B cell populations induced T cells to actually co-express IL-10 with IFNγ, IL-17A, or FoxP3 (**Figure 2E**). We show that the HC DP memB cells markedly increased the frequency of T cells that co-expressed IL-10 and IL-17A versus the HC DN, HC TIM-1+ or the MS DP memB cells. Representative FACS plots of these co-cultures in which analyte gating was based on FMOs, are shown in **Figure 2F**. Together these data suggest that the TIM-1+ and DP memB cells employ distinct modes of T cell regulation in the HC that are dysfunctional in the MS-derived cells.

Next, we determined whether the different memB cell subset co-cultures differed in accumulated secreted cytokines (**Figure 2G**). MS DN memB cultures were found to contain significantly greater IFNγ and TNFα than the HC DN memB cultures which were produced at similar levels in the HC TIM-1+ or HC DP cultures. For the MS-derived subsets, the TIM-1+ or DP memB cultures contained significantly less TNFα (both) and IFNγ (DP memB) than the DN memB cell cultures. In assessing IL-17A, although the HC and MS DN memB cultures produced similar levels of IL-17A, only the HC DP memB cell-stimulated T cells exhibited a significantly reduced amount of IL-17A compared to the respective donor DN memB cell cultures. Similarly, only the HC DP memB cell cultures showed less IL-6 production compared to HC TIM-1 and DN memB cell cultures, suggesting that, unlike the MS-derived cells, the low IL-6 production by HC DP memB cells contributes to low Th17 differentiation.

Lastly, as these B-T co-cultures aim to compare the capacity of HC vs MS B cells to affect T cell stimulation and cytokine production, we reasoned that the autologous T cells from HCs vs patients could have potentially disparate starting activation or polarization states. Thus, we established these B-T co-cultures to utilize the same allogeneic, 3^rd^ party HC CD4+ T cell in all assays with the different donor memB cell subsets. This raises the possibility that MHC disparity could be a major determinant in the outcome of these assays. While it would be unusual that all such reactivity would be active only with the MS-derived B cells, we examined how this allogeneic disparity may affect the B-T assay. Using the most T cell activativing B cell subset, we established co-cultures with HC or MS DN memB cells and the allogeneic CD4+ T cells under conditions of no supplemental anti-CD3 stimulus, soluble anti-CD3 or soluble anti-CD3/anti-CD28 (**Figure 2H**). Without additional anti-CD3 stimulus, T cell proliferation in cultures with DN memB cells providing potential allogenic stimulation was similar to the T cell proliferation in cultures that only contained T cells. Upon adding soluble anti-CD3, there was significant but low level T cell proliferation in co-cultures containing the HC or MS DN memB cells, with no proliferation in T cell only cultures. Notably, the CD4+ T cells proliferated without APCs in response to soluble anti-CD3/anti-CD28 stimuli, a response that was strongly magnified by providing the HC or MS DN memB cells. Thus, while the B-T cultures do have both anti-CD3 and potential allogeneic stimuli, the anti-CD3 and co-stimulation from the memB cells appears to drive proliferation in these cultures.

### The HC TIM-1+TIGIT+ DP memB cells are more suppressive than the MS-derived cells

**Figure 3:**
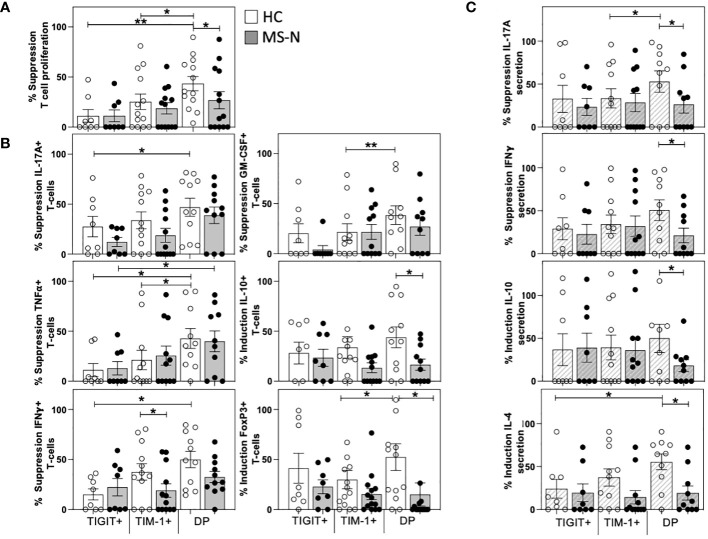
TIM-1+TIGIT+ DP memB cells are a highly suppressive from HC and not in MS. The capacity of TIM-1+, TIGIT+, and DP memB cells to suppress T cell activation mediated by the same donor-derived DN memB cells was determined in three-way B-B-T co-cultures. The CD4+ T cells were all from the same HC donor. HC or MS-derived memB cell subsets were shown to have distinct effects on the suppression of **(A)** T cell proliferation, **(B)** Intracellular T cell expression of IL-17A, IFNγ, GM-CSF, TNFα, IL-10 and FoxP3. **(C)** Cytokine secretion in day 6 co-culture supernatants. Suppression was calculated as: (1-(%cell division of CD4+ T cells in the triple co-cultures containing autologous DNmemB and TIM-1/TIGIT memB subsets divided by % cell division of CD4+ T cells with the same DN memB cells only)*100). All MS donors were being treated with Natalizumab. Significance was determined by paired One-Way Anova and HC vs. MS comparison by unpaired One-way Anova, Sidak’s correction for multiple comparisons was used for all analyses. (*p<0.05, **p<0.005).

After determining the T cell activating properties of the different TIM-1+/-TIGIT+/- memB cells (B-T assay), we examined whether these different B cell populations could suppress T cell activation. To determine suppressive activity, we designed what we call a B-B-T assay that tests whether the HC- and MS-N-derived TIM-1+, TIGIT+, or DP memB cells can inhibit DN memB cell-mediated activation of the same allogeneic HC T cells in a triple component co-culture at a ratio of 1:1:2 in the presence of suboptimal soluble anti-CD3. In this B-B-T assay, the CD4+ T cell proliferation was measured in DN memB B-T cultures and used to normalize the proliferation and cytokine activity of T cells cultured with DN memB cells together with each of the TIM-1+, TIGIT+ or DP memB cell populations. Neither the HC nor MS-derived TIGIT+ memB cells were able to reduce the level of T cell activation (**Figure 3A**). Thus the TIGIT+ memB cells do not appear to have any Breg activity. In contrast, both the HC and MS TIM-1+ memB cells exhibited low-level inhibition of T cell proliferation, although they did not significantly differ from the activity exhibited by the TIGIT+ memB cells. Uniquely, the HC DP memB cells exhibited a highly inhibitory function inducing suppression of T cell proliferation more than the HC TIGIT+ or HC TIM-1+ memB cells. Importantly, DP memB cells from HC subjects also exhibited a much greater capacity to suppress T cell proliferation than the MS DP memB cells (**Figure 3A**).

We next examined how the different donor sources of the memB populations affected the frequency of cytokine-producing T cells in these triple B-B-T co-cultures. Again, the HC and MS-derived TIGIT memB cells showed no differences in their capacity to modulate cytokine-expressing T cells. The MS TIM-1+ memB cell cultures produced more T cells expressing IFNγ (**Figure 3B**) than T cells cultured with the HC TIM-1+ memB cells. The MS and HC DP memB cells showed a greater ability to suppress TNFα expression than the autologous TIGIT+ memB cells. However, only the HC DP memB induced greater inhibition of IL-17A, IFNγ, and TNFα (**Figure 3B**) than the HC TIGIT+ memB cells and greater suppression of GM-CSF and TNFα than the HC TIM-1+ memB cells. HC DP memB cells also had a greater ability to induce FoxP3 expression compared to TIM-1+ memB cells. Importantly, in comparing the activity of the HC vs MS DP memB cells, not only did the HC DP memB cells give greater suppression of T cell proliferation, they also induced a significantly greater frequency of T cells expressing IL-10 and FoxP3 than the MS DP memB cells (**Figure 3B**). Thus, the MS-derived DP memB cells were less able to suppress proliferation and were less able to induce expression of IL-10 or FoxP3 in the co-cultured T cells. Further, although the HC and MS TIM-1+ memB cells showed no difference in their capacity to suppress T cell proliferation, MS TIM-1+ memB cells were less able to suppress IFNγ expression in T cells.

Quantification of the cytokines secreted in these triple co-cultures (**Figure 3C**) confirmed the inhibition of intracellular cytokine expression (IL-17A and IFNγ) and further demonstrated that the HC DP memB cell cultures markedly increased IL-4 and IL-10 secretion compared to the MS DP memB cell cultures. Thus, comparing the two populations that were non-inflammatory in the B-T assay, i.e., the TIM-1+ memB and DP memB cells, only the TIM-1+TIGIT+ DP memB cells exhibited a highly suppressive, Breg-like activity, and this activity was impaired in MS.

### Increased DP memB cell frequency is associated with improving expanded disability status scale in anti-CD20 treated MS patients

**Figure 4:**
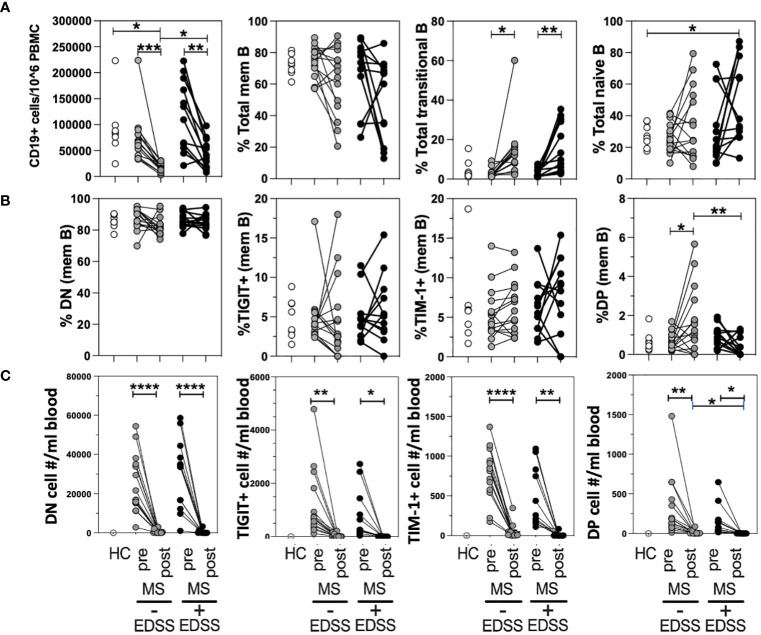
DP memB cell frequency is increased in anti-CD20 treated RRMS patients with improving vs worsening EDSS. PBMCs from HCs and age/sex-matched RRMS patients that differed in response to anti-CD20 therapy (increasing versus decreasing EDSS after treatment) were examined for B cell subset frequency and absolute cell number. **(A)** The total numbers of B cells per 10^6 PBMCs and the percentages of memory, transitional, and naïve B cells were examined. **(B)** Within the memB cell population (CD19+CD20+CD27+) the frequency of TIM-1+, TIGIT+. DN, and DP cells was determined. **(C)** The number of cells per mL blood was calculated for each memB subset for pre- and post anti-CD20 samples using blood donation volume and PBMC cellularity. Significance was determined by paired One-Way Anova, comparison for pre vs. post treatment in each donor and by unpaired One-way Anova with Sidak’s correction for multiple comparisons between donors. (*p<0.05, **p<0.005, ***p<0.0005, ****p<0.0001).

Although anti-CD20 therapy is highly efficacious, it has been found that roughly twenty percent of patients do not show lasting improvement which can be reflected in their EDSS score (45). To determine if a positive response to anti-CD20 was associated with changes in the frequency of any TIM-1+/-TIGIT+/- memB cell subset, we performed a retrospective longitudinal study. We selected thirteen non-responder RRMS patients who had given blood before and after anti-CD20 therapy (Ocrevus) but did not respond to therapy as indicated by an increase in EDSS score. We also identified fourteen responder RRMS patients that were matched for sex, age, and EDSS at the start of treatment, that had donated pre- and post- anti-CD20 therapy, but responded to treatment with a decrease in EDSS. The patients in each cohort were also matched for the other DMTs that they had been taking before donating blood on the day they initiated anti-CD20 therapy as there was no drug washout period per standard clinical guidelines (these data on DMT and EDSS are provided in **Supplementary Tables 2**, **3**). The different samples were examined for total CD19+ cell number (out of total PBMCs) and for frequencies of memory, transitional (CD38+CD24^hi^), and naïve B cell populations within all CD19+ B cells (**Figure 4A**). The frequencies of DN memB, TIM-1+ memB, TIGIT+ memB and TIM-1+TIGIT+ DP memB subsets within the pool of memory B cells were also examined (**Figure 4B**). The cellular representation of each memB subset per mL of blood was also determined (**Figure 4C**). As expected, all anti-CD20 treated patients showed a striking depletion of total CD19+ cells and an increased frequency of transitional B cells that has been described by others (17), while non-responder patients showed an increased frequency of naïve B cells compared to HC. Interestingly, when examining the TIM-1 and TIGIT expressing memB cell subsets, the only population to show significant differences in frequency between responders and non-responders was the DP memB subset which was increased in the responder MS patient group that did not show increased EDSS after anti-CD20 treatment. In addressing whether this result was also recapitulated when the actual subset cell number/mL blood was determined, we again found that there was a significant increase in the number of DP Bregs when the post-treatment samples were compared between responder vs non-responder patients (**Figure 4C**).

### Differential gene expression analysis shows MS DP memB cells have increased expression of genes associated with activation and survival, and decreased Breg function

**Figure 5:**
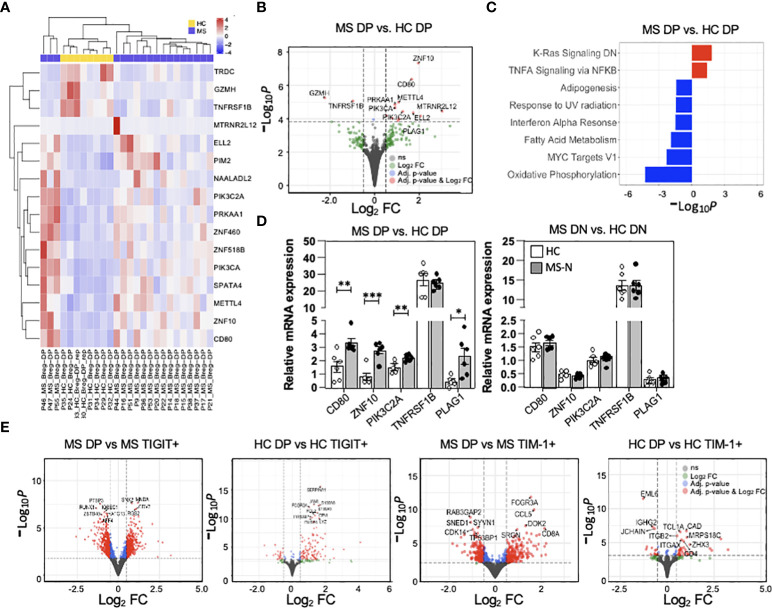
MS DP memB cells show unique transcriptional differences associated with altered stimulatory function. **(A)** Heatmap showing the scaled expression level for statistically significant genes at an adjusted p-value < 0.1. The heatmap has been annotated by disease status. **(B)** Volcano plot showing the global transcriptional changes in MS (Natalizumab) vs. HC DP memB cells and denoting specific genes. The points highlighted in red are genes that have an adjusted p-value < 0.1 and an absolute log_2_ fold change (FC) > 0.5. Points in blue have an adjusted p-value < 0.1 and a log_2_ FC < 0.5 and points in green have an adjusted p-value > 0.1 and a log_2_ FC > 0.5. Grey points are non-significant. The dashed lines correspond to the cutoff values of log_2_ FC and adjusted p-value. Positive log_2_ fold changes indicate increased expression in MS samples. **(C)** Gene enrichment analysis of MS vs HC DP memB cells using the hallmark gene set, -Log_10_ adjusted p-values have been multiplied by 1 or -1 according to the direction of the change. Positive values represent enrichment in MS compared to HC (red) and negative values represent enrichment in HC compared to MS (blue). **(D)** qPCR validation of five genes that were identified to distinguish MS vs. HC DP memB cells in the RNA-Seq analysis in part **(A, B)**, using DP memB (left) and DN memB (right) cells from 6 RRMS (natalizumab treated) and 6 HCs. **(E)** Volcano plots showing global transcriptional changes between MS or HC DP memB cells and their autologous TIGIT+ or TIM-1+ memB cells. The adjusted p-values and color coding are the same as discussed in part **(B)** above. Positive log_2_ fold changes indicate increased expression in DP memB cells. (*p<0.05, **p<0.005, ***p<0.0005).

As the MS-derived DP memB cells (from natalizumab treated patients) functionally differed in their capacity to suppress proliferation and IFNγ and IL-17A cytokine expression or induce IL-10 and FoxP3 expression in the co-cultured T cells, we next examined the transcriptional landscape of MS vs HC-derived DP memB cells. Having planned to co-examine function and gene expression, an aliquot of 1000 cells was set aside for RNA isolation upon the initial FACS-sorting of each population. Gene expression analysis of the low input RNA-Seq datasets identified roughly two sets of genes showing differential expression between HC and MS cells (**Figure 5A**), with specific genes showing high log_2_ fold changes (volcano plot, **Figure 5B**). Genes with increased expression in the MS DP memB cells included those found in signatures of antigen presentation and inflammation (CD80), and accelerated B cell proliferation and survival (METTL4, PIK3C2A). Due to our limited sample size for this clinically based study, only 16 genes met our adjusted p-value threshold of 0.1 for statistical significance, and the sub-analysis correlating gene expression with *in vitro* function could not be performed. The difference in the expression of these 16 genes between the MS and HC samples is shown in **Supplementary Figure 1**. The most significantly up-regulated gene in MS DP memB cells (**Figure 5B**) is ZNF10 which regulates apoptosis and glycolytic activity (46). In contrast, the TNFRS1B gene, encoding TNFR2 which is expressed by IL-10-producing Bregs, was one of the most significantly downregulated genes in MS DP memB cells relative to HCs (31). GSEA analysis (**Figure 5C**) indicated that the MS Bregs are enriched in pathways of B cell activation and inflammatory responses (KRAS and TNFα signaling). In contrast, the HC DP memB DGE analysis indicated enrichment for metabolic and survival pathways (oxidative phosphorylation, adipogenesis, and IFNα pathways) (**Figure 5C**) (47–50).

To validate the low-input RNA-Seq results, DP and DN memB cells were subsequently isolated from an additional 6 HCs and 6 RRMS (Natalizumab) samples and a subset of DGE genes were measured by qPCR. MS-derived DP memB cells showed increased relative mRNA expression of ZNF10, CD80, PIK3C2A, and PLAG1 (which was just below our significance cut-off), representing four of the five tested genes compared to the HC DP memB cells (**Figure 5D**). A similar analysis of the HC vs. MS DN memB cells showed no differences in the expression of these genes. To examine the relationship between the TIGIT+ and TIM-1+ memB subsets and their DP memB cell counterparts within MS or within HC samples at the transcriptional level, the respective low input bulk RNA-Seq analyses were performed (**Figure 5E**). Overall, between the HC TIM-1+ or HC TIGIT+ memB and the HC DP (Breg) population, 37 and 132 genes, respectively, were differentially expressed. Between the MS TIM-1+ or MS TIGIT+ memB and the MS DP (Breg) population 418 and 912 genes, respectively, were differentially expressed. For the HC DP memB cells, while the majority of genes showed increased expression compared to the TIGIT+ or the TIM-1+ memB cells, a number of unique genes were also down-regulated in comparison to the TIM-1+ memB cells. In contrast to the HC population, more genes were differentially expressed in the comparisons of MS DP memB against the TIGIT+ or the TIM-1+ MS memB cells, with comparable numbers of up vs. downregulated genes (GSEA analysis and gene expression analyses of these data are presented in **Supplementary Tables 4**, **5**). Full differential expression results for each contrast including apeglm shrunken Log_2_Fold changes, annotation from the Ensembl database v.94, and DESeq2 normalized counts for all samples included in the comparisons are given in **Supplementary Tables 4, 5**. The GSEA analysis (**Supplementary Table 5**) using the Hallmark gene set includes the number of genes in the gene set (setSize), the enrichment score normalized across all gene sets (NES), which indicates the extent of the enrichment of these genes, the position in the ranked list at which the maximum enrichment score occurred for that gene set (Rank), and the subset of genes that contributes the most to the enrichment score (core_enrichment).

## Discussion

B cell-depleting therapies are highly efficacious for the treatment of MS, but near-total depletion of B cells can lead to an increased risk of infections and malignancy and hamper the efficacy of vaccinations (51). Thus, our goal was to identify mechanisms that delineate between pathogenic effector B cells and anti-inflammatory regulatory B cell populations in order to identify selective immunotherapeutic agents that only target disease-associated effector B cells while sparing those with regulatory/anti-inflammatory properties (52). In this study, we characterized the regulatory potential of memory B cells (CD19+CD20+CD27+) differentially expressing TIM-1 and TIGIT in PBMCs from MS patients compared to those from healthy controls. We demonstrated five main observations: First, DN memB cells consistently induced the highest level of T cell proliferation and proinflammatory cytokine expression, whereas the TIM-1+ TIGIT+ DP memB cells displayed the most regulatory activity, exhibiting the ability to suppress T cell proliferation and IL-17A, IFNγ and TNFα production. Second, the DN memB cells from MS patients were more pro-inflammatory than those from HC while the MS DP B cells were less suppressive than those from HC. Third, MS patients who responded to anti-CD20 therapy exhibited an increased frequency of DP memB cells that was not seen in samples from non-responder MS patients. Fourth, the DP memB cells from MS patients were less able to induce T cell co-expression of IL-10 with FoxP3 or IL-17. Lastly, transcriptional analyses indicated that MS DP memB cells showed an increased expression of genes involved in inflammation and B cell activation as compared to HC DP memB cells.

Several studies have shown that TIM-1 expression on CD19+ B cells confers specific regulatory activity and is fundamental for the prevention of autoimmunity (29, 53). TIM-1 expression and signaling in B cells enables optimal IL-10 production and clearance of apoptotic cells, while the lack of TIM-1 on B cells promotes the generation of CD4+ T cells expressing IFNγ and IL-17A with reduced expression of IL-10 (29). Human and mouse TIM-1+ B cells are highly enriched for IL-10 production (54, 55), and dysregulation in this population is associated with inflammatory disease in humans (53, 56). CD24hi CD39hi memB cells that exhibit high expression of TIM-1 and other immune checkpoint molecules including TIGIT and PD-L1, were found to modulate dendritic cell function, suppress Th1 and Th17 T cell responses, and produce IL-10 (57). TIM-1 signaling was also shown to be required for TIGIT expression in B cells and essential for B cell-mediated regulation and maintenance of CNS homeostasis (29, 56, 57). Further, MS-derived memB cells have been shown to be impaired for both TIGIT expression and ability to suppress IL-17A-secreting follicular T helper cells, suggesting that TIGIT may play a prominent role in B cell regulatory activity (58).

As additional immune checkpoint receptors are known to be expressed on B cell subsets, we asked whether healthy donor B cells co-expressing TIM-1 and TIGIT represent an optimal Breg population compared to TIM-1+TIGIT- memB cells. Here, we found that the TIM-1+ TIGIT+ DP memB subset not only showed the lowest ability to support T cell proliferation and cytokine production, but also was more suppressive than the TIM-1+ or the TIGIT+ memB cells. In fact, our data suggest that the TIGIT+ (TIM-1-) memB cells have no regulatory activity. Thus as a previous report showed regulation by TIGIT+ B cells (58), we posit that the regulatory activity ascribed to these TIGIT+ B cells was likely attributable to those TIGIT+ cells that co-express TIM-1. Altogether, these data again underscore that TIM-1 expression appears to be fundamental for Breg function and that TIM-1 and TIGIT co-expression marks a novel human, highly inhibitory, memory Breg population.

The transcriptional differences between the TIM-1+TIGIT+ HC and MS Bregs suggest that the MS DP memB cells are in an altered state of activation. The functional assays indicated that the MS DP Bregs are less suppressive than those from HCs, while transcriptional analyses indicated that the MS DP Bregs are poised to induce extrinsic T cell activation while exhibiting unique activation of intrinsic cell growth and survival pathways. The genes overexpressed in MS DP memB cells include CD80, a T cell costimulatory molecule that is increased in B cells by B cell activating stimuli, GM-CSF, and disease activity, and functions to greatly augment T cell activation (10, 59, 60), although whether the increased CD80 mRNA translates into greater CD80 surface protein expression needs to be determined as CD80 expression is sensitive to post-transcriptional mechanisms that regulate its protein expression (61). In contrast, the prominent expression of ZNF10 in MS-derived DP memB cells is known to enhance intrinsic cell proliferation, cell cycle progression, germinal center B cell metabolic capacity, and prevent apoptosis via the activation of β-catenin and GSK3β phosphorylation (46, 62). Enhanced expression of PIK3CA in MS-derived DP memB cells, the P110 subunit of PI3K, is known to be fundamental for B cell activation, differentiation, survival, and Breg development (63). MS DP memB cells are also enriched for KRAS and NF-κB pathways which are augmented by TNFα signaling and lead to increases in BCR induced survival and activation. KRAS is fundamental in early B cell development and late B cell maturation as B cell-specific deletion of KRAS impairs B cell development and BCR-induced activation via the Raf-1/MEK/ERK pathway in mature B cells (47). Similarly, rela^-/-^ and p50 knockout animal models indicated a role of NF-κB in B cell maturation and proliferation in response to BCR ligation (48). These pathways highlight ways in which MS DP memB are poised to exhibit enhanced survival and potentially enhanced T cell activation.

In contrast, genes that were markedly reduced in the MS DP memB cells include those involved in regulatory and protective immune responses. The low input RNA-Seq analyses showed that the MS DP memB cells exhibited lower expression of TNFRFS1B which encodes TNFR2, a marker of memory B cells that regulate via their capacity to produce IL-10 (31, 64); and Granzyme H (GMZH), which is a serine protease related to caspase-activating Granzyme B that exhibits immunoregulatory effects in T cells in autoimmune conditions (65) (**Figure 5**). As GzmH expression could not be detected in two thirds of the MS qPCR validation samples, we were unable to confirm its reduced expression in MS DP Bregs (data not shown), and whether the delay in RNA sample solubilization between the RNA solubilization after FACS sorting viable cells vs validation sorting cells directly into RNA isolation buffer remains to be tested. Conversely, the HC DP memB cell gene expression profile indicates the cells are in an augmented state of oxidative phosphorylation, which is associated with increased B cell IL-10 production and Breg function in mouse models (50); have elevated IFNα activation which can promote B cell resistance to apoptosis during inflammation (66); and have upregulated fatty acid metabolism and adipogenesis metabolic pathways suggesting the HC DP Bregs may have the ability to fulfil higher energy demands than the MS Bregs (49). Overall, the transcriptional landscape suggests that the MS-derived DP memB cells are enhanced for antigen-presenting activities and contain reduced regulatory and metabolic-response mechanisms. Despite our small sample size and limited statistical power, this exploratory and hypothesis-generating gene expression study revealed informative trends in MS B cell transcription that will be explored in larger datasets in the future.

The ability of B cell depletion therapy to limit new disease relapse occurs primarily via the targeting of antibody-independent pathogenic B cell mechanisms (8, 67). Following anti-CD20 treatment, the reconstituting B cell lineage is highly enriched in transitional B cells and strongly diminished in memory and naïve B cells (17), while the frequency of plasmablasts (per ml of blood) remains unchanged in MS patients (17). In our study, comparing responders and non-responders to anti-CD20 therapy, we also observed that the reconstituting B cells exhibited a marked increase in the relative frequency of transitional B cells while memory and naïve B cell populations were not significantly different. Interestingly, within the memB cell pool the frequency of the TIM-1+ TIGIT+ DP memB cells only increased in those patients identified as responders to therapy (**Figure 4B**). Thus, these data suggest that an increased presence of the regulatory DP memB cells post-treatment may contribute to the efficacy of anti-CD20 therapy in responding patients. This would be in accord with the findings that reconstituting memory B cells following anti-CD20 therapy are more anti-inflammatory with increased IL-10 production and less pro-inflammatory with reduced TNFα and GM-CSF production (8, 10, 68). Therefore, we propose one mechanism of response to anti-CD20 therapy is via a preferential restoration of functional TIM-1+ TIGIT+ Bregs during B cell lineage reconstitution.

In summary, we found that memory B cells differentially expressing TIM-1 and TIGIT supported different levels of T cell proliferation and selective effects on the expression of IFNγ, IL-17A, or FoxP3 with or without IL-10 co-expression, to add to the list of conditions that appear to distinctly regulate Th1 and Th17 such as glutamine metabolism, TCR signal strength, and immune checkpoint molecules (69–71). Altogether, these data support the conclusion that the DP memB cells are a bonafide Breg population. Thus, these data identify a novel regulatory memory B cell population that can be isolated via surface co-expression of TIM-1 and TIGIT and which is dysfunctional in MS.

## Data availability statement

The data presented in the study are deposited in the GEO repository, accession number GSE261258.

## Ethics statement

The studies involving humans were approved by The Institutional Review Board (IRB) of Mass General Brigham. The studies were conducted in accordance with the local legislation and institutional requirements. The participants provided their written informed consent to participate in this study.

## Author contributions

JV: Data curation, Investigation, Methodology, Writing – original draft, Writing – review & editing. BK: Data curation, Investigation, Methodology, Visualization, Writing – original draft, Writing – review & editing. FvG: Data curation, Investigation, Methodology, Writing – review & editing. JC: Data curation, Investigation, Methodology, Writing – review & editing. ZL: Data curation, Investigation, Methodology, Writing – review & editing. AJ: Data curation, Formal analysis, Methodology, Software, Writing – review & editing. EB: Data curation, Formal analysis, Methodology, Software, Writing – review & editing. SS: Data curation, Formal analysis, Methodology, Software, Writing – review & editing. YH: Data curation, Methodology, Writing – review & editing, Formal analysis, Validation, Visualization. RK: Investigation, Writing – review & editing, Data curation, Methodology, Project administration. TC: Resources, Writing – review & editing, Investigation. VK: Conceptualization, Resources, Supervision, Writing – original draft, Writing – review & editing, Investigation, Visualization. HW: Conceptualization, Supervision, Writing – original draft, Writing – review & editing, Funding acquisition, Resources. CB-A: Conceptualization, Investigation, Methodology, Project administration, Supervision, Visualization, Writing – original draft, Writing – review & editing.

## Supplementary material

Supplementary Figure 1 DGE between HC and MS-derived DP memB/Bregs. Visual representation of the extent of differences in expression in genes differing between the ex vivo MS and HC DP Breg cells. Abundance (log_2_ normalized counts) of the top 16 ranked genes varying between MS vs. HC DP memB cells (Bregs) from the DEA ranked by adjusted p-value with significance at p<0.1.

Supplementary Table 4 MS DP memB cells show increased transcriptional differences in comparison to autologous TIM-1 and TIGIT memB cells. Full differential gene expression and GSEA analyses of the comparisons performed between HC DP memB vs MS DP memB, HC DP memB vs HC TIGIT memB, HC DP memB vs HC TIM-1+ memB, MS DP memB vs MS TIGIT memB, and MS DP memB vs MS TIM-1 are given. The differential expression results include apeglm shrunken Log2Fold changes, annotation from the Ensembl database v.94, and DESeq2 normalized counts for all samples.

Supplementary Table 5 The GSEA analysis using the Hallmark gene set includes the number of genes in the gene set (setSize), the enrichment score which indicates the extent of the enrichment of these genes, the enrichment score after it is has been normalized across gene sets (NES), the position in the ranked list at which the maximum enrichment score occurred for that gene set (Rank), and the subset of genes that contributes the most to the enrichment score (core_enrichment). The GSEA analysis for the HC DPmemB vs MS DP memB is in **Figure 5**.
