# Supplementary figures and images for: Human regulatory memory B cells defined by expression of TIM-1 and TIGIT are dysfunctional in multiple sclerosis
Source: Front Immunol. 2024 Apr 30;15:1360219. doi: 10.3389/fimmu.2024.1360219 (PMC11091236; doi:10.3389/fimmu.2024.1360219)

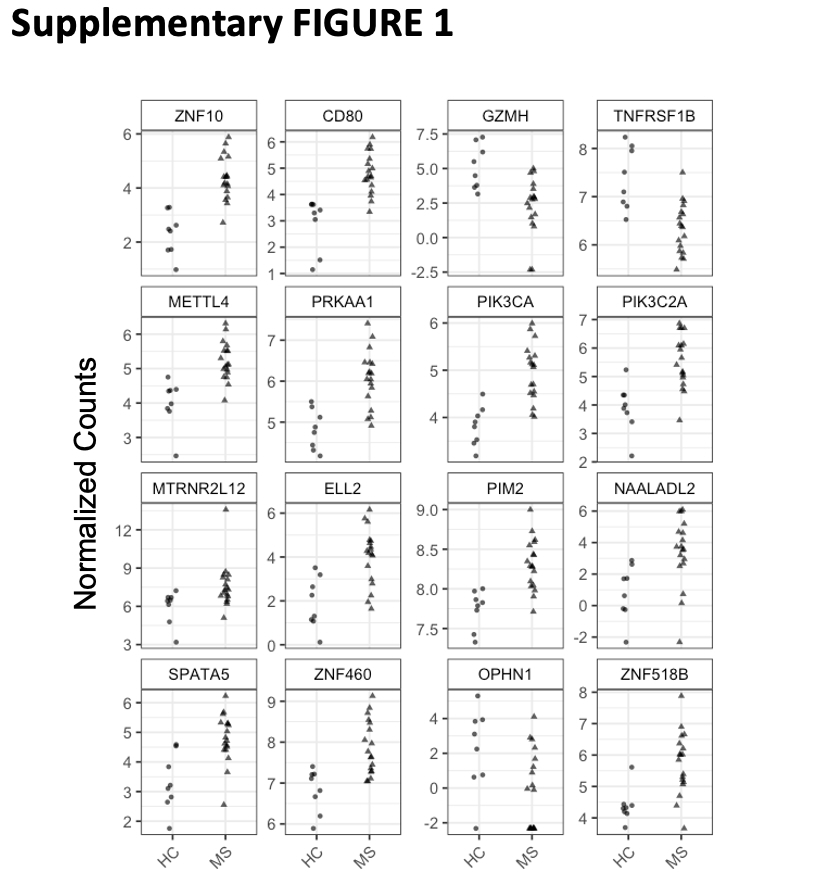

Supplement: Supplementary Figure 1 — DGE between HC and MS-derived DP memB/Bregs. Visual representation of the extent of differences in expression in genes differing between the ex vivo MS and HC DP Breg cells. Abundance (log2 normalized counts) of the top 16 ranked genes varying between MS vs. HC DP memB cells (Bregs) from the DEA ranked by adjusted p-value with significance at p<0.1. [file DataSheet_1.zip › Supplementary Figure 1.JPEG]

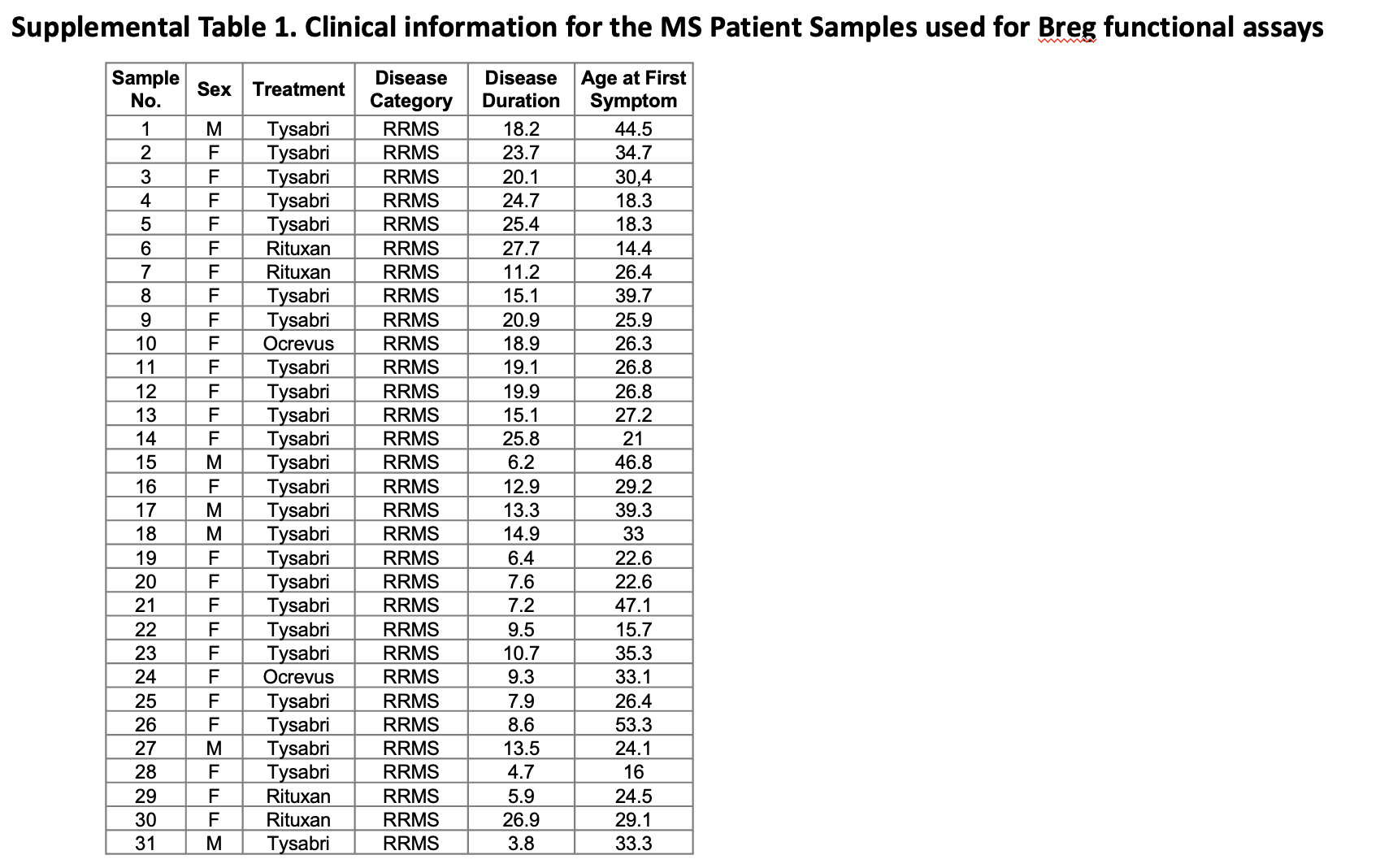

Supplement: Supplementary Figure 1 — DGE between HC and MS-derived DP memB/Bregs. Visual representation of the extent of differences in expression in genes differing between the ex vivo MS and HC DP Breg cells. Abundance (log2 normalized counts) of the top 16 ranked genes varying between MS vs. HC DP memB cells (Bregs) from the DEA ranked by adjusted p-value with significance at p<0.1. [file DataSheet_1.zip › Supplementary Table 1.JPEG]

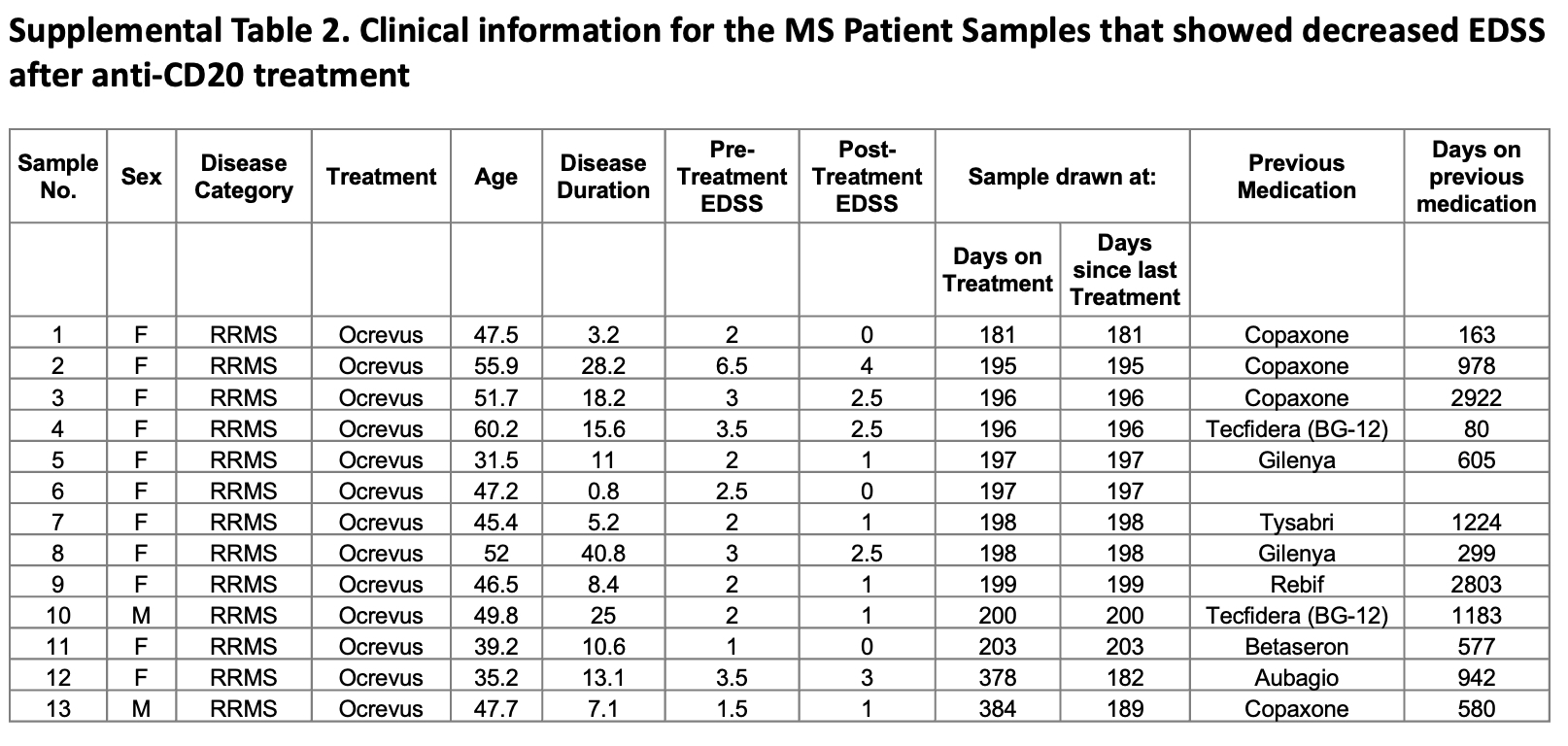

Supplement: Supplementary Figure 1 — DGE between HC and MS-derived DP memB/Bregs. Visual representation of the extent of differences in expression in genes differing between the ex vivo MS and HC DP Breg cells. Abundance (log2 normalized counts) of the top 16 ranked genes varying between MS vs. HC DP memB cells (Bregs) from the DEA ranked by adjusted p-value with significance at p<0.1. [file DataSheet_1.zip › Supplementary Table 2.JPEG]

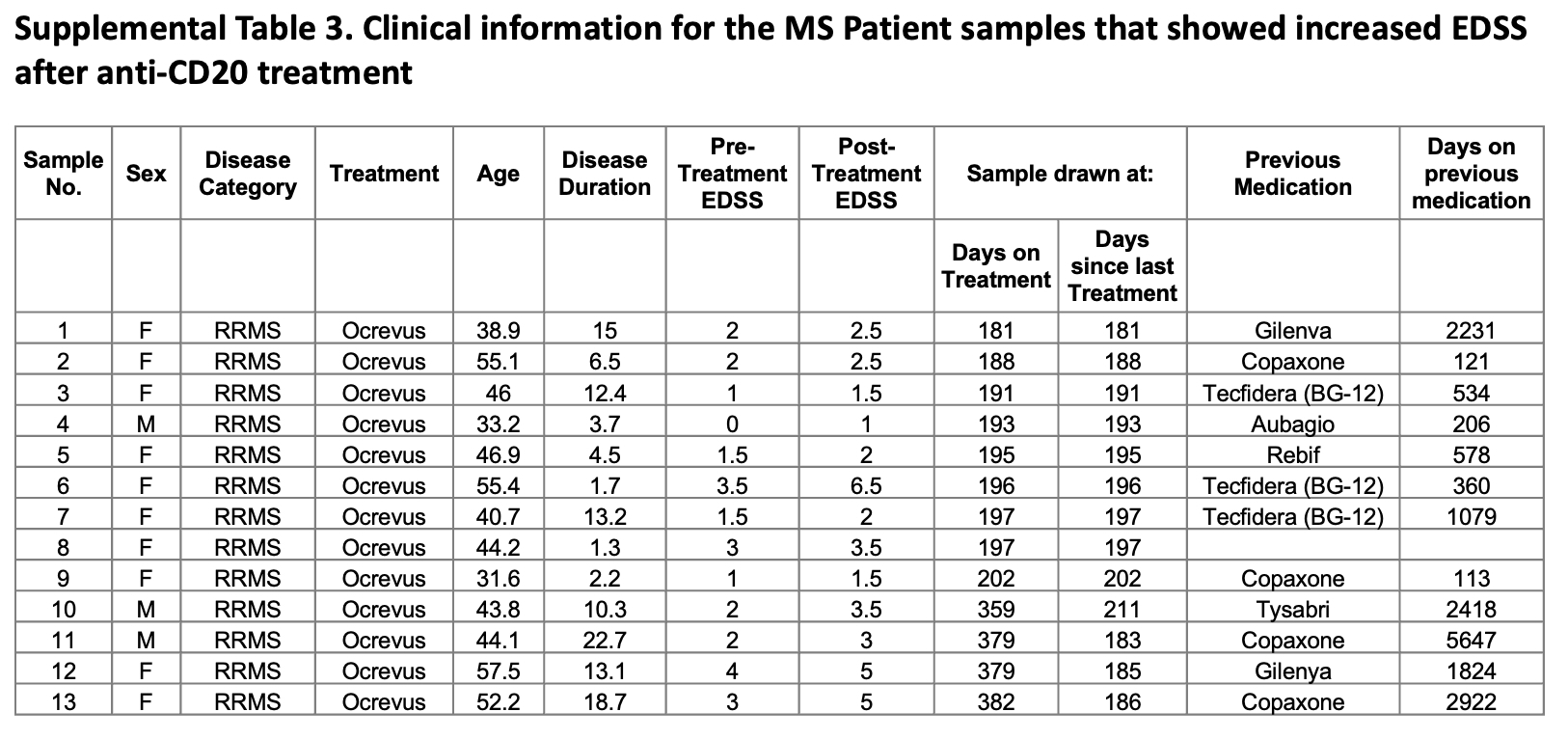

Supplement: Supplementary Figure 1 — DGE between HC and MS-derived DP memB/Bregs. Visual representation of the extent of differences in expression in genes differing between the ex vivo MS and HC DP Breg cells. Abundance (log2 normalized counts) of the top 16 ranked genes varying between MS vs. HC DP memB cells (Bregs) from the DEA ranked by adjusted p-value with significance at p<0.1. [file DataSheet_1.zip › Supplementary Table 3.png]
